# seq-ImmuCC: Cell-Centric View of Tissue Transcriptome Measuring Cellular Compositions of Immune Microenvironment From Mouse RNA-Seq Data

**DOI:** 10.3389/fimmu.2018.01286

**Published:** 2018-06-05

**Authors:** Ziyi Chen, Lijun Quan, Anfei Huang, Qiang Zhao, Yao Yuan, Xuye Yuan, Qin Shen, Jingzhe Shang, Yinyin Ben, F. Xiao-Feng Qin, Aiping Wu

**Affiliations:** ^1^Center for Systems Medicine, Institute of Basic Medical Sciences, Chinese Academy of Medical Sciences and Peking Union Medical College, Beijing, China; ^2^Suzhou Institute of Systems Medicine, Suzhou, Jiangsu, China; ^3^School of Life Science and Technology, China Pharmaceutical University, Nanjing, China; ^4^School of Pharmacy, Health Science Center, Xi’an Jiaotong University, Xi’an, China

**Keywords:** mouse, RNA-Seq, immune cell, deconvolution, tumor, machine learning

## Abstract

The RNA sequencing approach has been broadly used to provide gene-, pathway-, and network-centric analyses for various cell and tissue samples. However, thus far, rich cellular information carried in tissue samples has not been thoroughly characterized from RNA-Seq data. Therefore, it would expand our horizons to better understand the biological processes of the body by incorporating a cell-centric view of tissue transcriptome. Here, a computational model named seq-ImmuCC was developed to infer the relative proportions of 10 major immune cells in mouse tissues from RNA-Seq data. The performance of seq-ImmuCC was evaluated among multiple computational algorithms, transcriptional platforms, and simulated and experimental datasets. The test results showed its stable performance and superb consistency with experimental observations under different conditions. With seq-ImmuCC, we generated the comprehensive landscape of immune cell compositions in 27 normal mouse tissues and extracted the distinct signatures of immune cell proportion among various tissue types. Furthermore, we quantitatively characterized and compared 18 different types of mouse tumor tissues of distinct cell origins with their immune cell compositions, which provided a comprehensive and informative measurement for the immune microenvironment inside tumor tissues. The online server of seq-ImmuCC are freely available at http://wap-lab.org:3200/immune/.

## Introduction

High-throughput RNA sequencing (RNA-Seq) has now been widely applied in mouse models to study the transcriptome of different disease conditions, such as tumors (1), infections (2), and autoimmune inflammation (3), and this has led to the rapid accumulation of enormous RNA-Seq data in Sequence Read Archive (SRA). Transcriptomal analyses are traditionally focused on characterizing the biological functions under variable physiological or pathological conditions at the molecular level, namely in a gene-centric view (4). Gene module and pathway or network based annotation further expands the understanding of RNA-Seq data into the pathway-centric view (5) or the network-centric view (6).

In recent years, a few computational methods have been developed to extract cellular information, especially the tissue immune contexture from transcriptomal data (7–9). The basic hypothesis under these methods is that the gene expression profile in tissues is a linear combination of the gene expressed from all of the included cell types. According to their derived and applied data platforms, these methods can be divided into three types, namely, microarray-derived and microarray-applied models, microarray-derived and RNA-Seq-applied models, and RNA-Seq-derived and RNA-Seq-applied models. In microarray-derived models, several machine learning methods have been reported, including elastic net regularization (Elastic net) (10), linear least square regression (LLSR) (11), quadratic programming (QP) (12), and support vector regression (SVR) (13). Among these models, the SVR based method has been proven with good robustness and precision in both human and mouse samples (13, 14). Furthermore, to estimate the immune cell compositions from the sequencing data, some strategies have been adopted to use the RNA-Seq data with a microarray-derived model (10, 15, 16). Li et al. tried to remove the batch effect between different platforms with ComBat, which was first developed to adjust batch effects between microarray and RNA-Seq data (15). Trajanoski et al. characterized the intratumoral immune cell proportions by transforming RNA-Seq data into microarray-like data using a cubic smoothing spline with four degrees of freedom (16). Altboum et al. reported a computational method, digital cell quantification, to infer the proportion of 213 immune cells directly with microarray based training data (10). Up to now, only a few deconvolution methods derived from RNA-Seq data have also been applied. DeconRNASeq was the first framework for predicting the cellular content from RNA-Seq data although there is still no real training data to predict the immune cell proportion (17). Recently, a computational model with a RNA-Seq reference profile, named EPIC was also developed by Gfeller et al. to estimate the proportion of immune and cancer cells from human tumor transcriptomal data (18). These models have been well used to investigate the cellular microenvironment in diseased tissues, such as tumors (19). However, a model to predict immune cell compositions from increasing mouse RNA-Seq data is still lacking.

Here, a computational model, named seq-ImmuCC, was developed to predict the constitution of 10 immune cells from the RNA-Seq data of mouse tissues. After collecting and filtering available mouse RNA-Seq data from SRA, a signature gene matrix, including 162 genes specific for 10 major immune cells, was constructed. Subsequently, six machine learning methods were compared in the same signature gene matrix. The testing results indicated that the SVR- and LLSR-based models tended to achieve better performance in both simulated and experimental data. Furthermore, to validate the rationality of the computational model across different platforms, four combinations with microarray- or RNA-Seq-based training or testing data were compared. In general, models with consistent training and testing data types had better performances, while models with discordant data types achieved worse results, although they are still useful for some datasets.

With the computational advantage of the seq-ImmuCC model, we built an atlas of immune cell compositions in normal and tumor mouse tissues. In total, 27 normal tissues and 18 tumor tissues were included and the relative compositions of 10 major immune cell types were inferred for each mouse tissue. The comprehensive immune cell profiles provided not only the baseline of steady state immune cell proportions for most of the normal tissues, but also the measurement for highly complex and diverse immune microenvironments of various mouse tumor models.

## Materials and Methods

### Schematics of Methodology Development

Four major steps have been taken to construct the seq-ImmuCC model: (1) data collection and filtering: raw RNA sequencing data collected from SRA were preprocessed. Samples that can be clearly grouped were kept for later analysis; (2) signature gene selection: the differentially expressed genes (DEGs) in each of the cell types were achieved with voom (20) in the “limma” package, and then the genes that were highly expressed in the non-hematopoietic and tumor tissues were removed; (3) algorithm selection: six machine learning methods were compared for their performance in synthetic data and experimental data; and (4) model evaluation: the determined model was evaluated with enriched immune cells, simulated complex tumor data, and experimental flow cytometry data.

### Dataset and Preprocessing

Three different datasets were scanned from the public SRA database using the “R” package in SRAdb (21). In total, 358 enriched immune cells, 2,435 normal tissues, and 2,016 tumor tissues were downloaded from SRA. Datasets that were profiled on Illumina sequencing platforms with spots larger than 10 M were kept. Finally, 286 immune cell samples, 527 normal tissues, and 686 tumor samples were retained for later analysis (Table S1 in Supplementary Material). The raw fastq format of the RNA-Seq data was preprocessed using FastQC and trimmatic, and then mapped to the mouse mm10 genome using STAR. The read counts were calculated using HTSeq. Specially, the read counts of each V, D, and J gene segments in both the T cell and B cell receptors were merged. Finally, a quantile normalization was performed on each sample. The scripts for data preprocessing can be downloaded from the ImmuCC web server^1^ or the Github site.^2^

### Signature Gene Matrix Construction

According to the lineage tree of immune cells, RNA-Seq data of the terminally differentiated immune cells were scanned from the public database and only those cell types with enough sequencing data were kept for our analysis. In total, 286 RNA-Seq datasets of 10 immune cells, including B cells, CD4 T cells, CD8 T cells, macrophages, monocytes, neutrophils, mast cells, eosinophils, dendritic cells, and natural killer cells, were selected according to sample clustering and PCA analysis. Cell types that can be precisely grouped and have a specific expression on their marker genes were kept for later analysis. The DEGs in each immune cell were calculated using voom. Genes with an adjusted *P* value < 0.05 and log2-fold change > 2 were considered to be significant DEGs. Furthermore, genes that are highly expressed in both non-hematopoietic tissues and tumor tissues were filtered out as described in our previous work (14). To further minimize the gene number, genes with maximum read counts < 100 across all of the immune cells were filtered out. Finally, all of the genes that were left were ordered by decreasing fold changes and the top 20 signature genes in each cell type were selected to construct the signature gene matrix.

### Assessment of Algorithms

To determine which algorithm is appropriate for the seq-ImmuCC model, the performances of six machine learning methods, including ridge regression, least absolute shrinkage and selection operator (LASSO), Elastic net, LLSR (11), QP (12), and SVR (13), were assessed with both simulated and experimental data. The method for simulated data construction and experimental design were described in our previous work (14). In terms of the simulated data, we first made a random expression profile for the immune mixture with known compositions. Then, this immune mixture was mixed with the expression profile of a tumor cell line sample with different concentrations, ranging from 0.1 to 100%. Pearson correlation coefficient (PCC) between the predicted proportions and the real input proportions were calculated. In terms of the experimental data, the proportions that were calculated with six different algorithms were compared to the observed proportions from flow cytometry.

### Model Comparison Across Microarray and RNA-Seq Platforms

To evaluate the reliability of model cross platforms, the training data and testing data from both the microarray and RNA-Seq platforms were combined into four groups, Array-Array (microarray-based training and microarray-based testing), Array-RNAseq (microarray-based training and RNA-Seq-based testing), RNAseq-RNAseq (RNA-Seq-based training and RNA-Seq-based testing), and RNAseq-Array (RNA-Seq-based training and microarray-based testing). PCC between the predicted immune cell compositions and the quantitative flow cytometry measurements were calculated.

### RNA-Seq Library Preparation

Mouse samples including those of the spleen (SP), bone marrow (BM), lymph node (LN), and peripheral blood mononuclear cell (PBMC) collected in our previous work (14) were used here for RNA-Seq. Briefly, RNA-Seq libraries were constructed after rRNA depletion using a NEBNext rRNA Depletion Kit (Human/Mouse/Rat) (NEB). The E6310L NEBNext Ultra RNA Library Prep Kit for Illumina (NEB, E7530S) (NEB) was used according to the manufacturer’s instructions and the cDNAs were sequenced with the Hiseq X10 platform (Illumina).

### Data Availability

RNA-seq data have been deposited in the ArrayExpress database at EMBL-EBI (www.ebi.ac.uk/arrayexpress) under accession number E-MTAB-6458. The rest of the data is available from the authors upon reasonable request.

## Results

### Overview of the seq-ImmuCC Model

**Figure 1:**
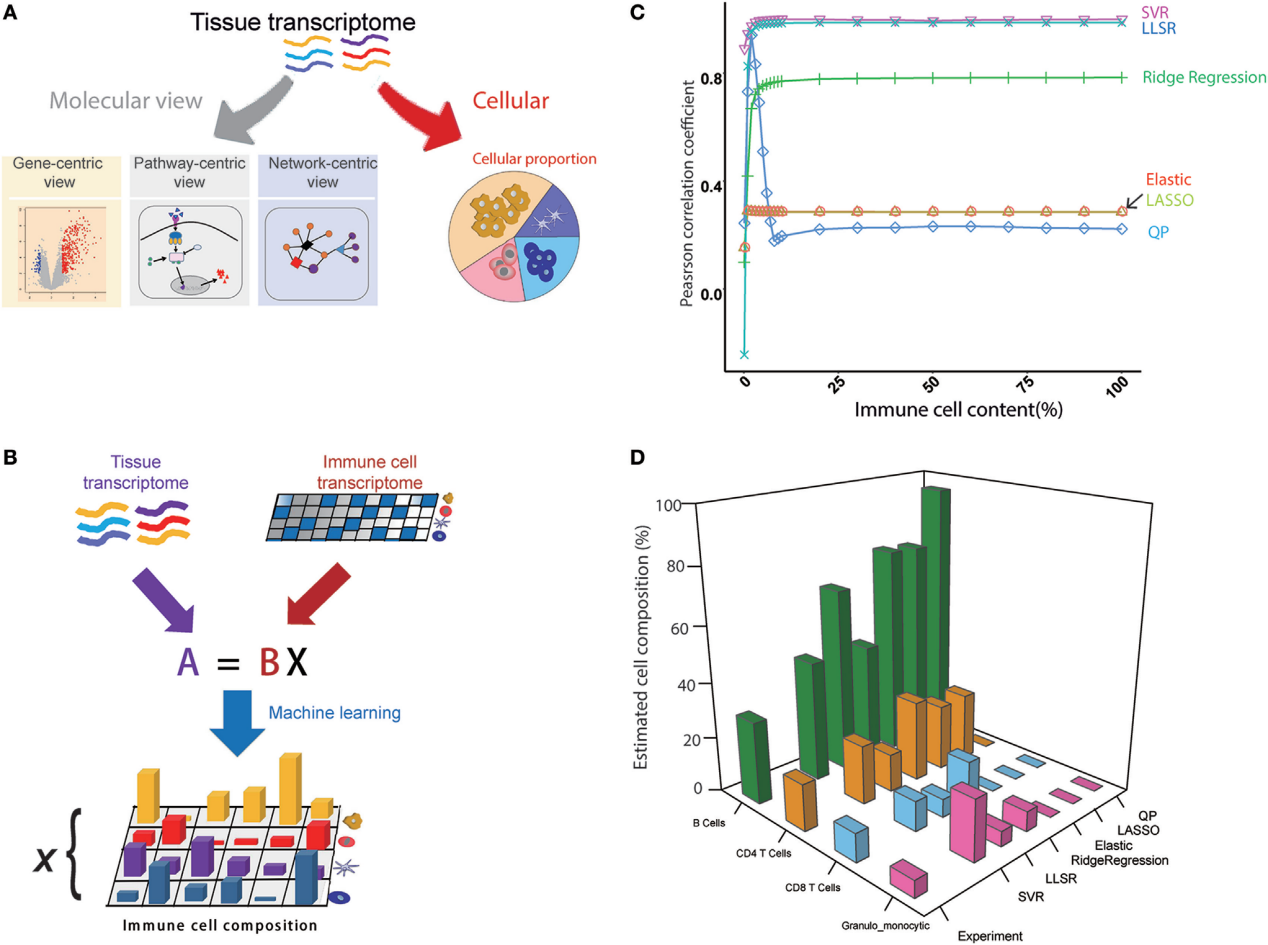
An overview of the seq-ImmuCC model. **(A)** Molecular and cellular views of the tissue transcriptome. **(B)** Schematics of the seq-ImmuCC model. **(C)** Comparison of six machine learning methods over the simulated data. **(D)** Comparison of six machine learning methods with the experimental data.

We assumed that the whole transcriptome is actually the comprehensive state of all of the genes expressed from different cell types within a mouse tissue, and then the cellular compositions can be deconvoluted from the transcriptome of the tissue (Figures 1A,B). The seq-ImmuCC model consists of four key steps (Figure S1 in Supplementary Material): (1) Sequencing data collection. The RNA-Seq data for each cell type were collected from the database and filtered. (2) Signature gene selection. The signature genes for each cell type were selected to construct the signature gene matrix. (3) Algorithm determination. The algorithm with the highest performance was used for the determined model. (4) Model evaluation. The model was evaluated with the simulated and experimental data.

### Signature Gene Selection

In total, 286 RNA-Seq datasets from the SRA database were collected. Then, after filtering with the expression of marker genes, 38 RNA-Seq datasets were retained to distinguish different immune cell types. Finally, 162 genes were selected as a signature matrix to cover 10 immune cells, namely, B cells, CD4 T cells, CD8 T cells, macrophages, monocytes, neutrophils, mast cells, eosinophils, dendritic cells, and natural killer cells. To test the distinguishing performance, the signature matrix was used to classify immune cells derived from different laboratories (Figure S2 in Supplementary Material). The clear grouping results indicated that the selected signature genes have an appropriate representativeness and high distinguishing ability.

### Model Building and Comparison

In order to obtain an accurate model, six machine learning methods were used to find the best way for predicting the immune cell composition with the same signature gene matrix, including LLSR (11), QP (12), LASSO, ridge regression, elastic net (10), and SVR (13). To compare the performance of the six models, PCC between the inferred proportions and the observed proportions was calculated. On a synthetic dataset with known immune cell compositions, both SVR- and LLSR-based models showed the highest PCC values. A significantly higher correlation was observed even when the proportion of tumor content reached 99% (Figure 1C). A relatively lower performance was shown in the ridge regression-based model with PCC as 0.78 when the tumor content ranged from 0 to 95% (Figure 1C). We further evaluated six models in the experimental dataset. As illustrated in Figure 1D, the relative fractions of four immune cell groups in the LN, namely, granulo-monocytic cells, CD4 T cells, CD8 T cells, and B cells, were calculated with different machine learning approaches. Consistent with the results in the simulated data, the proportions calculated with SVR were in good agreement with the flow cytometry results among B cells, CD4 T cells, and CD8 T cells. The relative abundance is also well matched to the measured results of LLSR and ridge regression methods, although there is a slight difference for a specific cell type.

### Model Evaluation

**Figure 2:**
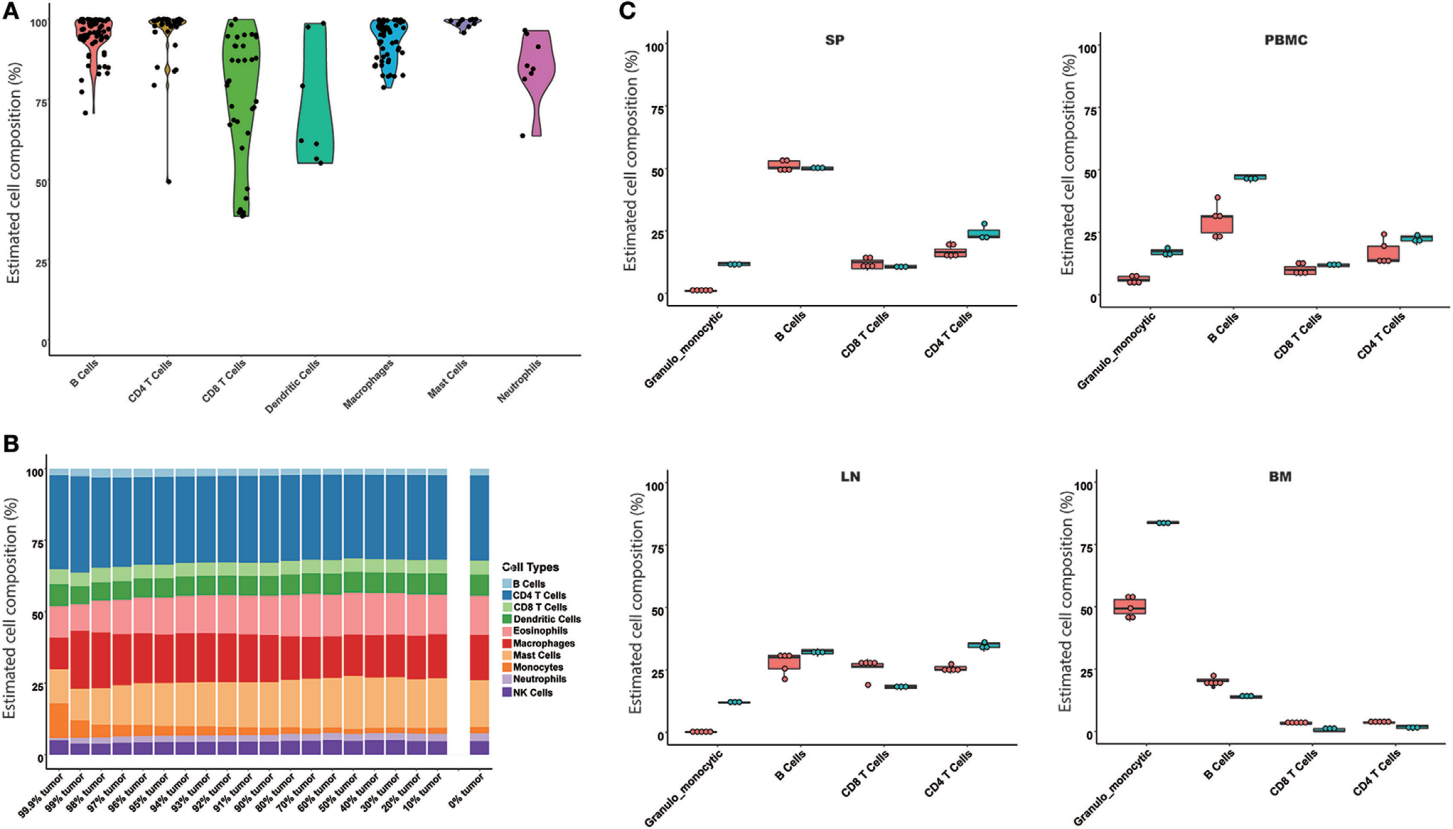
Evaluation of the seq-ImmuCC model in the simulated and experimental data. The performance of the model was evaluated in the enriched cell samples **(A)**, simulated tumor samples **(B)**, and measured results from flow cytometry **(C)**. Four immune cell types were compared in **(C)**, namely, granulo-monocytic cells, CD4 T cells, CD8 T cells, and B cells. Red: predicted results; green: flow cytometry results.

Based on the results of the comparison of the models, the SVR and LLSR models were suggested to predict immune cell compositions from RNA-Seq data, while only the SVR model was used as a representative model for further evaluation. The SVR model was evaluated on the simulated mixture samples, pure immune cell samples, and experimental tissues, respectively. For 245 samples of enriched single immune cells, we found the highest proportion in each sample was definitely consistent with the expected cell type, where the median predicted proportion was 85% (Figure 2A). Next, given the potential application of our model in heterogeneous tissues, a simulated tumor tissue with defined immune content (see Materials and Methods) was used to test its performance on complex tumor tissues. The predicted fractions were very consistent with the actual proportions even when the proportion of the tumor content reached 99.9%, which may provide solid evidence for its application on complex tissues (Figure 2B). Furthermore, we compared our model with the results from flow cytometry. As indicated in Figure 2C, the predicted results were all similar to the observed immune cell compositions across SP, PBMC, LN, and BM samples.

### Comparison of Microarray and RNA-Seq-Based Models

**Figure 3:**
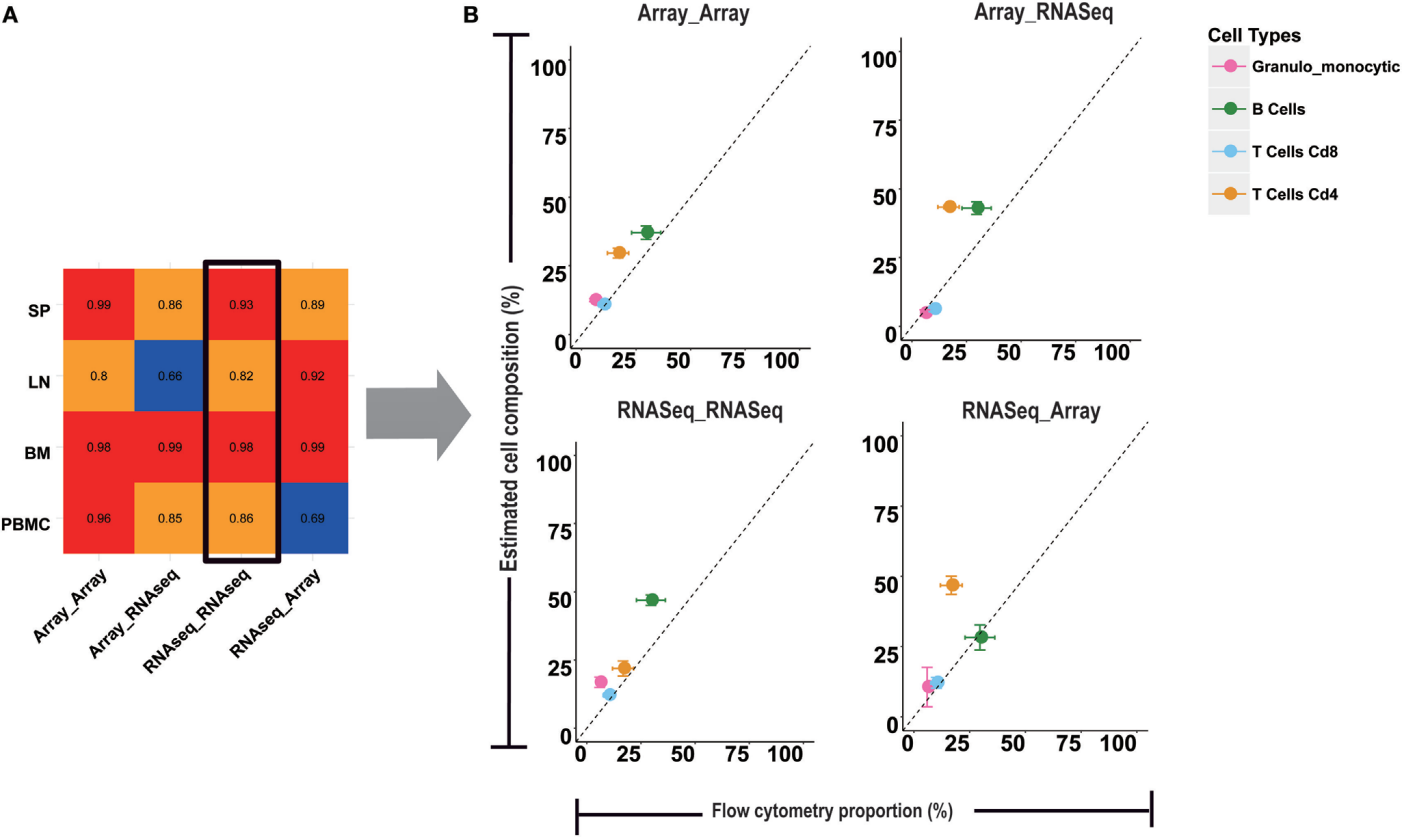
Comparison of microarray and RNA-seq based deconvolution models. **(A)** Four cross models with microarray or RNA-seq data as the training or testing input were compared. The value in the heatmap is the Pearson correlation coefficient between the results from four computational models and flow cytometry in four tissues. **(B)** The error bar plots are the comparison of the RNA-seq training and testing model with the flow cytometry for granulo-monocytic cells, CD4 T cells, CD8 T cells, and B cells in peripheral blood mononuclear cell.

Although some previous studies have already used microarray-based models to estimate immune cell compositions in RNA-Seq data (10, 15, 16), the reliability of the deconvolution model across microarray and RNA-Seq platforms is still unknown. Unlike microarray data, RNA-Seq data do not have a continuous distribution and usually tend to have a larger distribution of gene abundance. To examine the cross performances between microarray- and RNA-Seq-based models, four testing groups, named Array-Array, Array-RNAseq, RNAseq-RNAseq, and RNAseq-Array (see Materials and Methods for more details), were designed to predict the immune cell proportions in four types of immune samples, namely SP, LN, and BM, and PBMC (Figure 3; Figure S3 in Supplementary Material). As shown in Figure 3A, Array-Array outperformed the three other groups in SP, BM, and PBMC with a PCC larger than 0.9. In comparison to Array-Array, the RNAseq-RNAseq group presented a relative lower PCC ranging from 0.82 to 0.99. For the SP and PBMC samples, training data and testing data derived from the same platforms (Array-Array and RNAseq-RNAseq) tended to work better than the two cross groups (Array-RNAseq and RNAseq-Array). This observation suggested that the microarray-based model could be better used for microarray data, while the RNA-Seq-based model should be used for RNA-Seq data. Some bias may exist in some conditions when the microarray-based model is applied to RNA-Seq data, or reversed, although the general performance is still acceptable.

### An Atlas of Immune Cell Types in Normal Mouse Tissues

**Figure 4:**
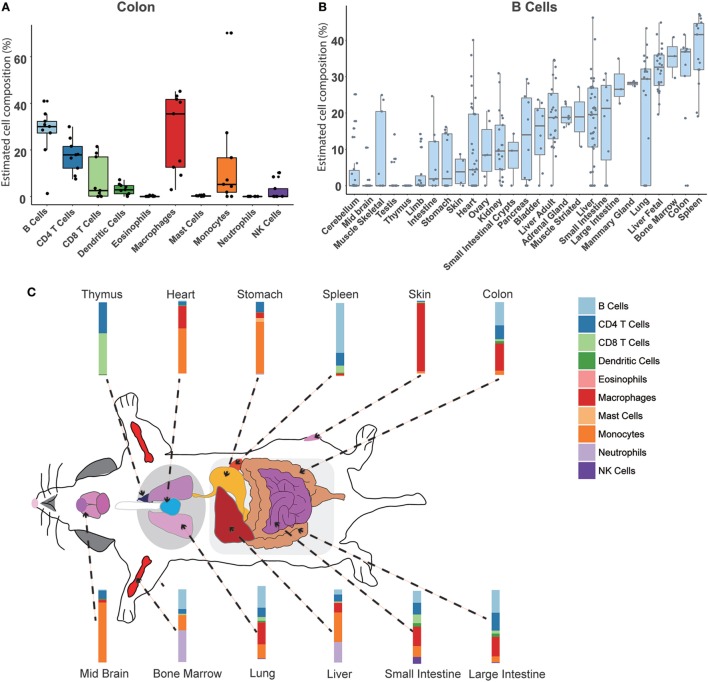
Atlas of immune cell compositions in normal mouse tissues. **(A)**. Inferred proportions of 10 immune cells in the colon. **(B)** Distribution of B cell proportion across 27 mouse tissues. **(C)** Immune cell fingerprint in 12 representative mouse tissues.

We used our model to systematically calculate the constitution of immune cells across different normal mouse tissues. In total, 27 normal tissue types profiled on the RNA-Seq platform were collected and evaluated (Table S1 in Supplementary Material). First, we could achieve the relative compositions of 10 immune cell types in a specific tissue. Taking the colon as an example (Figure 4A), the results indicated that the largest proportion is B cells (30 ± 18%), then about 25 ± 11% for macrophages, 10 ± 8% for CD4 T cells, and 10 ± 9% for CD8 T cells. Then, a specific immune cell type across multiple tissues could be estimated. As shown in Figure 4B, the distribution of B cell proportion among various tissues ranges from 0 to 50%. Consistent with our previous knowledge, the spleen has the highest relative proportion of B cells (40 ± 21%). The second highest proportion of B cells was in the colon tissue. In a gene-centric view, a high expression of IgA was found in the transcriptomal data of the colon, which may be associated with an enrichment of IgA production of plasma cells in the colon (Figure S4 in Supplementary Material). Interestingly, we noted that a relative higher B cell content was seen when compared among the fetal liver and the adult liver, which was consistent with the high expression level of IgM in the fetal liver (Figure S5 in Supplementary Material). Finally, a divergent immune content was observed among different normal mouse tissues (Figure 4C; Figures S6 and S7 in Supplementary Material). In the immune system organs, the abundance of corresponding immune cells was very consistent with our common sense. For example, as a primary lymphoid organ for the development of T cells, the thymus was mainly enriched with CD4 T cells and CD8 T cells. However, the relatively high proportion of neutrophils was observed in BM (50.85 ± 7.56%) and fetal liver (13.68 ± 5.65%), which is known to be the hematopoiesis organ at different stages of life. For most solid tissues, such as the skin, ovary, etc., the tissue immune microenvironment is mainly comprised of myeloid cells, including macrophages and monocytes. In the limb and skeletal muscle, there was a relatively higher abundance of mast cells (>20%). Different from other tissues, a higher amount of lymphocytes (B cells, T cells, and NK cells) in the intestines and mammary glands was also determined.

### An Atlas of Immune Cell Types in Mouse Tumor Tissues

**Figure 5:**
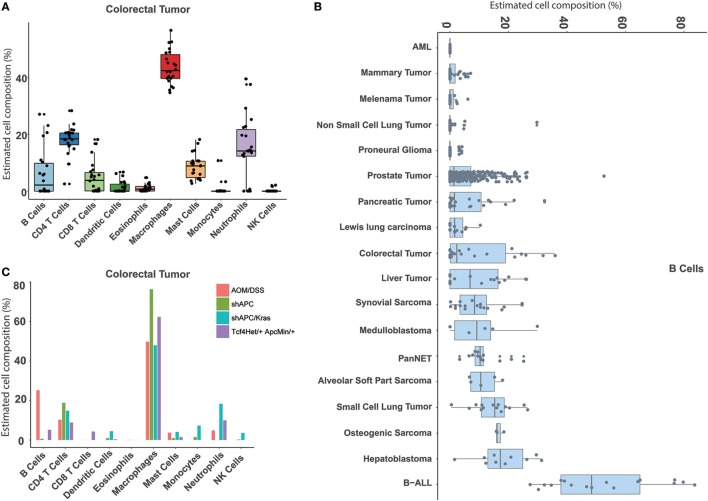
Atlas of immune cell compositions in mouse tumor tissues. **(A)** Inferred proportions of 10 immune cells in colorectal tumors. **(B)** Distribution of B cell proportion across 18 tumor types. **(C)** Comparison of the immune cell compositions in the same tumor type (colorectal tumors) with four different inducing models.

Similarly, our approach can also be used to estimate the immune cell proportions across different mouse tumor tissues. In total, 18 tumor types were collected and evaluated (Table S1 in Supplementary Material). First, compared to the normal tissue, a distinct immune signature was observed in the tumor sample (Figure S8 in Supplementary Material). For example, the major cell type in colorectal cancer is macrophage, whereas the most abundant one in the normal colon is B cell (Figures 4A and 5A). In addition, we found that different immune constitutions were observed among different tumor types. For example, a significant enrichment of leukocytes was observed in the leukemia samples. Acute myeloid leukemia was mainly constituted of neutrophils, whereas the dominant cell type in other types of solid tumors was macrophage (Figure 5B; Figure S9 in Supplementary Material). Next, the distribution of each immune cell across different tumor types was fully characterized. As shown in Figure 5B, the highest proportion of B cells was found in B-ALL, and similar proportions (~10%) of B cells were observed among hepatoblastoma and small cell lung cancer. As illustrated in Figure S9 in Supplementary Material, the highest proportion of CD8 T cells was observed in pancreatic neuroendocrine tumors (PanNET) as 25.02 ± 9.08%. Similarly, liver-derived tumors, including liver tumors and hepatoblastoma, also tended to infiltrate with a relatively higher level of CD8 T cells (13.93 ± 8.45%). Finally, with the seq-ImmuCC model, we could investigate the immune cell compositions in the same tumor type with different induced strategies. For each tumor type, variable strategies, such as chemically inducing, genetically modifying, etc., have been used to develop distinct tumor models. To investigate the difference of immune composition across different induced strategies, four different colorectal tumor models, including: AOM/DSS (Azoxymethane and dextran sodium sulfate induced model), shAPC, shAPC/Kras, and Tcf4Het/ + ApcMin/ + were used. As illustrated in Figure 5C, a significantly higher proportion of B cells was observed in the AOM/DSS-based model. Furthermore, compared with other groups, a relatively higher amount of neutrophils in shAPC/Kras was observed (Figure 5C).

### Online Web-Server

**Figure 6:**
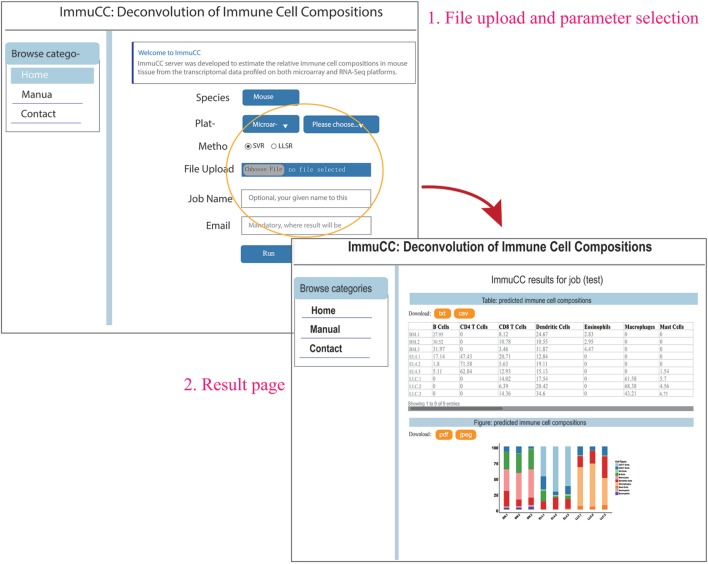
Online webserver for the deconvolution of immune cell compositions in mouse tissues. The webserver is available at http://wap-lab.org:3200/immune/.

An online webserver was implemented to infer the cellular proportions from the transcriptome by both microarray and RNA-Seq approaches, which is available at http://wap-lab.org:3200/immune/. As indicated in Figure 6, the samples profiled on various platforms, such as RNA-Seq approach, Affymetric mouse 430 2.0, Illumina MouseWG-6 v2.0 expression beadchip and Agilent Whole Mouse Genome Microarray 4 × 44K v2 were all available. Two machine learning methods, SVR and LLSR are presented as choices. The results include a table format file and a bar plot figure; if sample number is less than 10, the results will be presented on the same page and send *via* email.

## Discussion

In this study, we devised a computation model named seq-ImmuCC to infer the proportion of ten immune cells in mouse tissues from RNA-Seq data. To the best of our knowledge, this is the first deconvolution model that focuses on RNA-Seq data in mice. The performance of seq-ImmuCC has been validated in large and various types of independent datasets, including simulated data, public data and our own experimental data. The seq-ImmuCC model will provide an in-depth and accurate cell-centric view for transcriptomal data to monitor tissue infiltrating immune cells under various conditions. In order to better serve the scientific community, we have also developed an online user-interactive webserver.

Due to the absence of an RNA-Seq-based deconvolution model, some studies previously used microarray data based models to infer immune cell compositions from RNA-Seq data (10, 15, 16). However, there was still an open question of whether these models can be directly used across different transcriptomal platforms. Therefore, we conducted a systematic evaluation of the impact of the data platform on the performance by testing four groups of models across microarray data and RNA-Seq data. In general, better performances were observed in Array-Array and RNAseq-RNAseq based computational models in most cases, as expected. It is also worthy of noting that both Array-RNAseq and RNAseq-Array can still work well in certain conditions, which indicates the potential feasibility of using the model across microarray and RNA-Seq platforms.

Up to now, several machine learning methods have been proposed in the computational model to deconvolute immune cell compositions from transcriptomal data (7). Most of the previous researches including our own method (14), employed the linear regression model was employed. However, unlike microarray, RNA-Seq data do not have a continuous distribution and usually tend to have a larger distribution of abundance. In addition, the gene product in RNA-Seq data was usually exponentially amplified with PCR, which may further change the reads distribution. Therefore, a non-linear regression-based model may have a better performance in RNA-Seq data, which warrants more extensive work in future studies.

Using our developed seq-ImmuCC model, we can readily and reliably depict the constitution of major immune cells across different tissues or organs through the mouse transcriptomal data. Knowledge about the comprehensive immune cell constitution in various tissues would provide us an essential baseline to evaluate the potential local immune statues and will allow us to further characterize their difference of functionality in a molecular view. However, it should also be noted that tissue immune cell abundance could be influenced by many factors, including age, sex, and other physiological and environmental conditions (22). The variation of immune cell compositions among different mouse tissues presented here might reflect only part of the picture under given experimental conditions.

Predicting tumor-infiltrating immune cells is another important application of our model. With rapidly development, cancer immunotherapy is becoming a hot spot in basic immunological research and clinical investigation. Clinical trials have already indicated that tumor immune content could play a determinant role in disease prognosis and treatment selection (23, 24). Patients with high levels of intratumoral CD8 T cells while having low levels of regulatory T cells tend to have a better response to immune-based therapies (25). By extracting the valuable immune cell contexture information with our model, we can provide invaluable support to cancer immunological research with various mouse tumor models of human cancers. However, we have to caution that at the present time, our model has not been able to fully capture the tumor immune constitutions. For example, gamma delta T cells, which are now known to be important members of the immune system in fighting against tumors, were not included in our signature matrix. Therefore, further expansion and refinement of our model by putting more cell types into the composition matrix will be an important work in the near future.

## Ethics Statement

This study was carried out in accordance with the recommendations of the guidelines established by the Association for the Assessment and Accreditation of Laboratory Animal Care. The protocol was approved according to the policies and procedures for the Care and Use of Laboratory Animals in the Chinese Academy of Medical Sciences.

## Author Contributions

ZC, FQ, and AW conceived and designed the study. AH and QZ performed the experiments. ZC, LQ, FQ, and AW analyzed the data and results. LQ constructed the web server. XY and QS contributed to the data collection and data pre-procession. YY and JS contributed to the discussion and analysis of the studies. ZC, LQ, FQ, and AW wrote the paper. All authors have approved the final manuscript.

## Conflict of Interest Statement

The authors declare that the research was conducted in the absence of any commercial or financial relationships that could be construed as a potential conflict of interest.

## Footnotes

^1^http://wap-lab.org:3200/immune/ (Accessed: April 3, 2018).

^2^https://github.com/chenziyi/ImmuCC/blob/master/webserver/RNASeq_pipeline.sh (Accessed: April 3, 2018).

## Supplementary Material

Figure S1 Schematic of the ImmuCC model construction.

Figure S2 PCA of 162 selected genes in 286 enriched immune cell data.

Figure S3 Comparison of the RNA-Seq training and testing models with the flow cytometry for Granulo-monocytic cells, CD4 T cells, CD8 T cells, and B cells in the bone marrow, lymph nodes, and spleen.

Figure S4 Boxplot of IgA expression in 27 mouse tissues.

Figure S5 Heatmap for the expression profile of B cell-specific genes in the fetal liver and the adult liver.

Figure S6 Inferred proportions of 10 immune cells in 26 mouse tissues.

Figure S7 Distribution of CD4 T cells, CD8 T cells, macrophages, monocytes, neutrophils, mast cells, eosinophils, dendritic cells and natural killer cells proportion across 27 mouse tissues.

Figure S8 Inferred proportions of 10 immune cells in 17 mouse tumor tissues.

Figure S9 Distribution of CD4 T cells, CD8 T cells, macrophages, monocytes, neutrophils, mast cells, eosinophils, dendritic cells and natural killer cells proportion across 18 mouse tumor tissues.

Table S1 Immune cell data sets collected from the public database and the inferred immune proportion in both the normal tissue and the tumor tissues.
