# Supplementary material for: seq-ImmuCC: Cell-Centric View of Tissue Transcriptome Measuring Cellular Compositions of Immune Microenvironment From Mouse RNA-Seq Data
Source: Front Immunol. 2018 Jun 5;9:1286. doi: 10.3389/fimmu.2018.01286 (PMC5996037; doi:10.3389/fimmu.2018.01286)
Supplement: Figure S1 — Schematic of the ImmuCC model construction. [file data_sheet_2.PDF]

Supplementary Figure 1

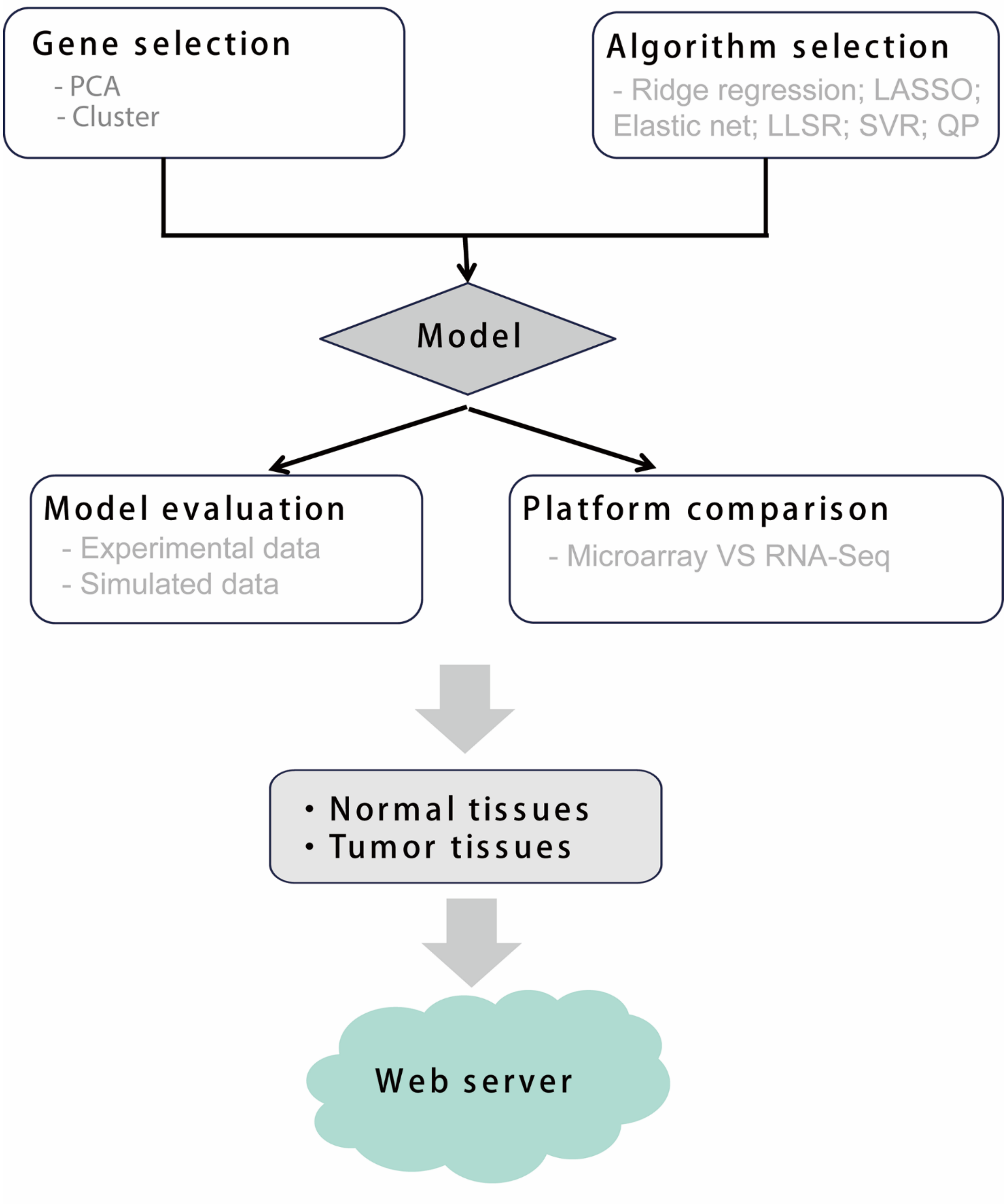

Supplementary Figure 2

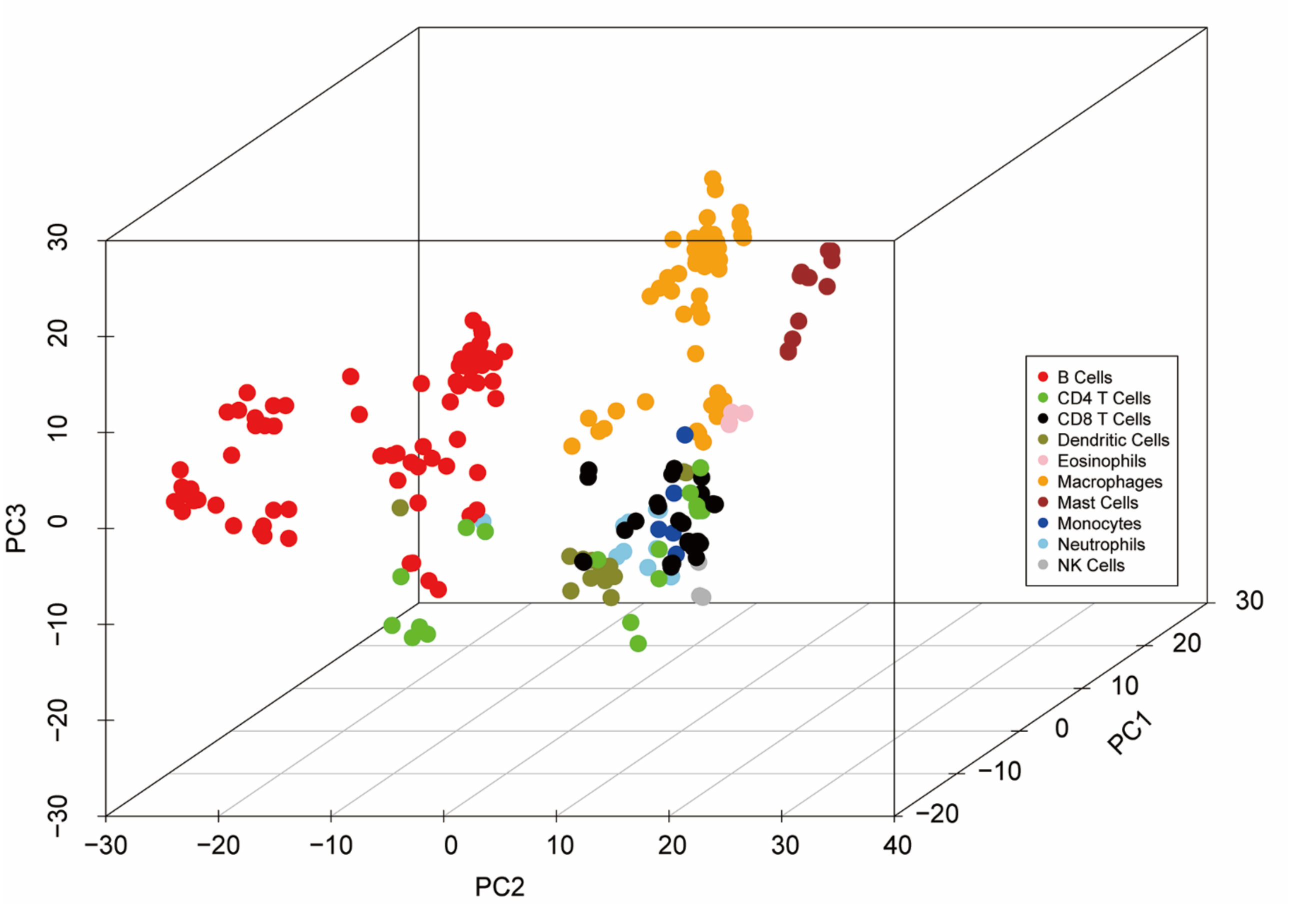

Supplementary Figure 3

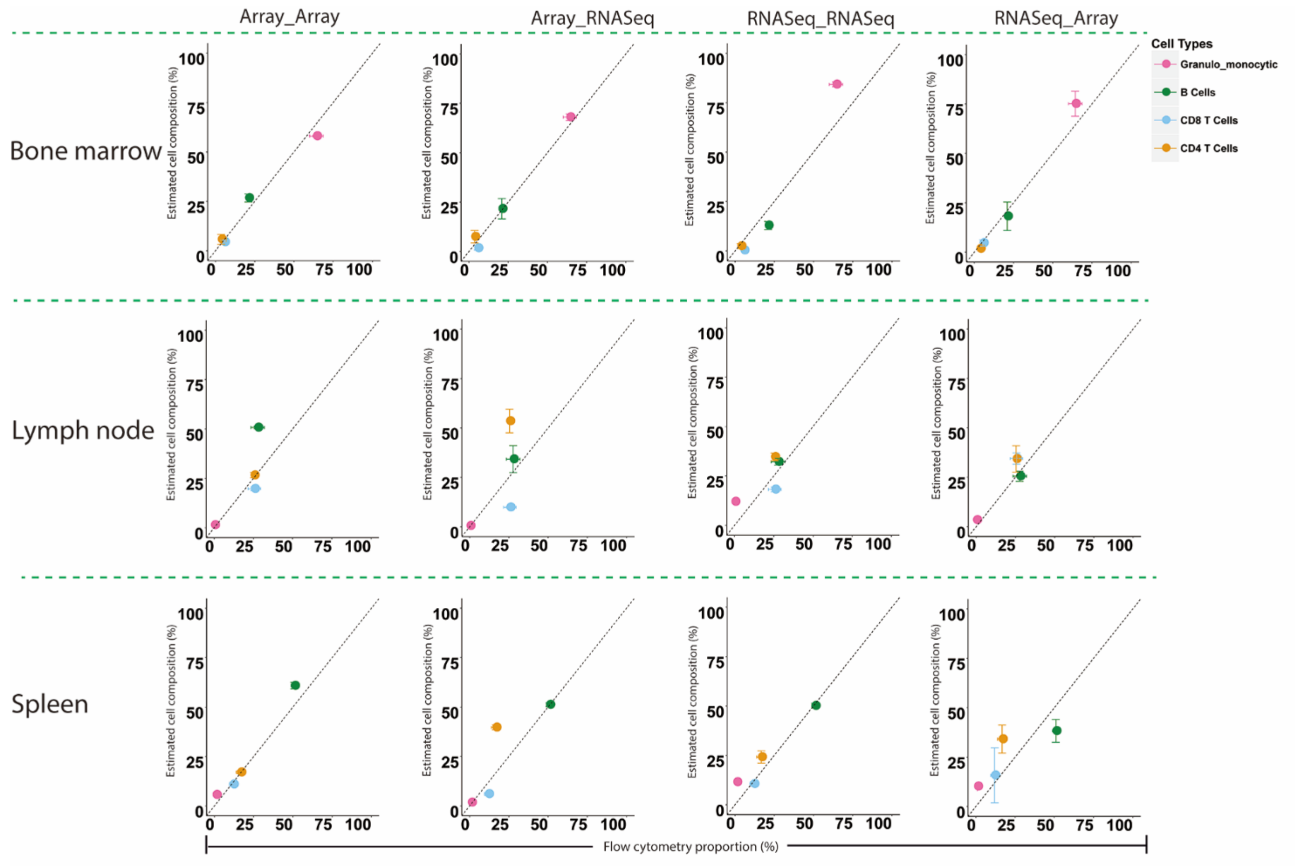

Supplementary Figure 4

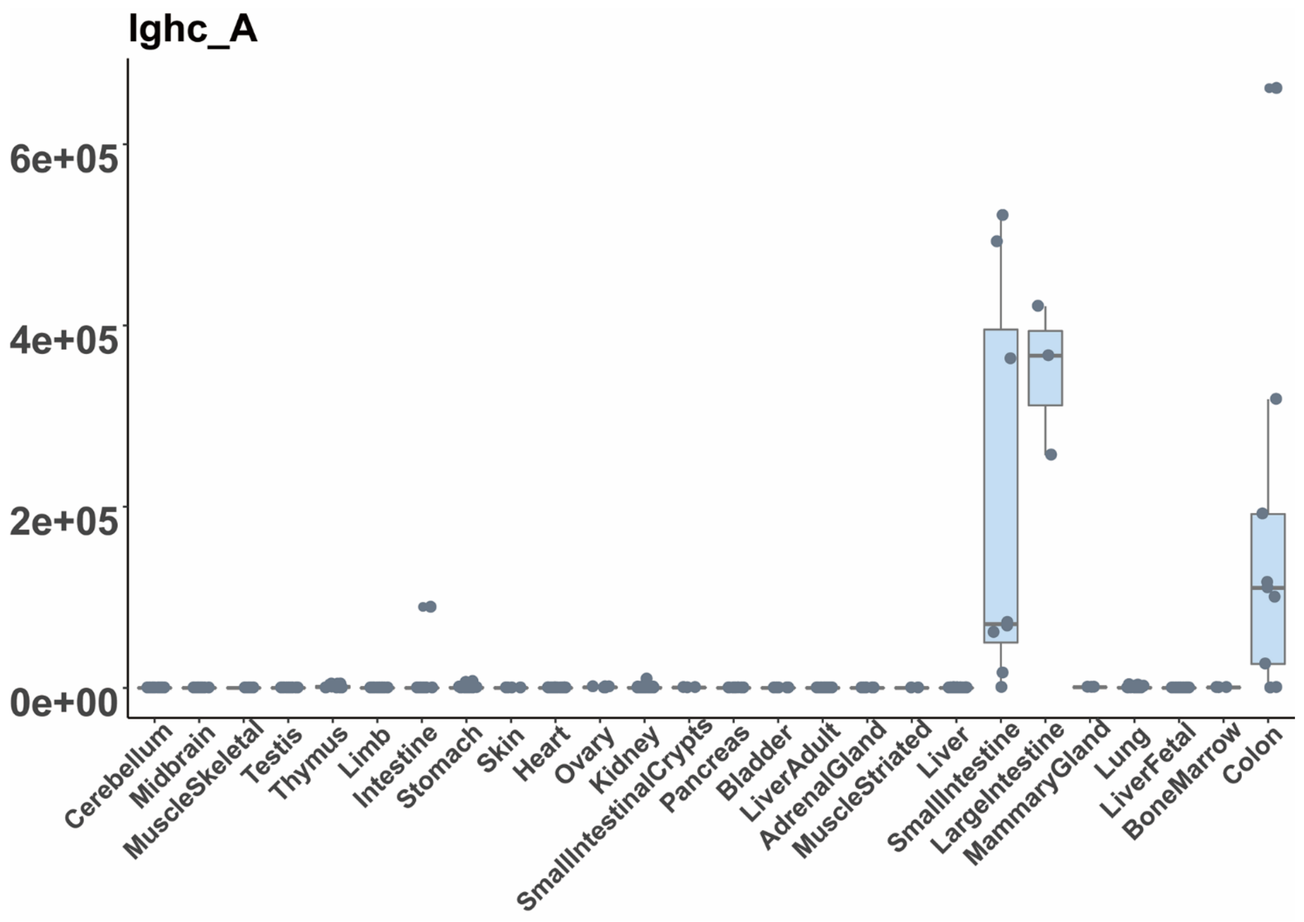

Supplementary Figure 5

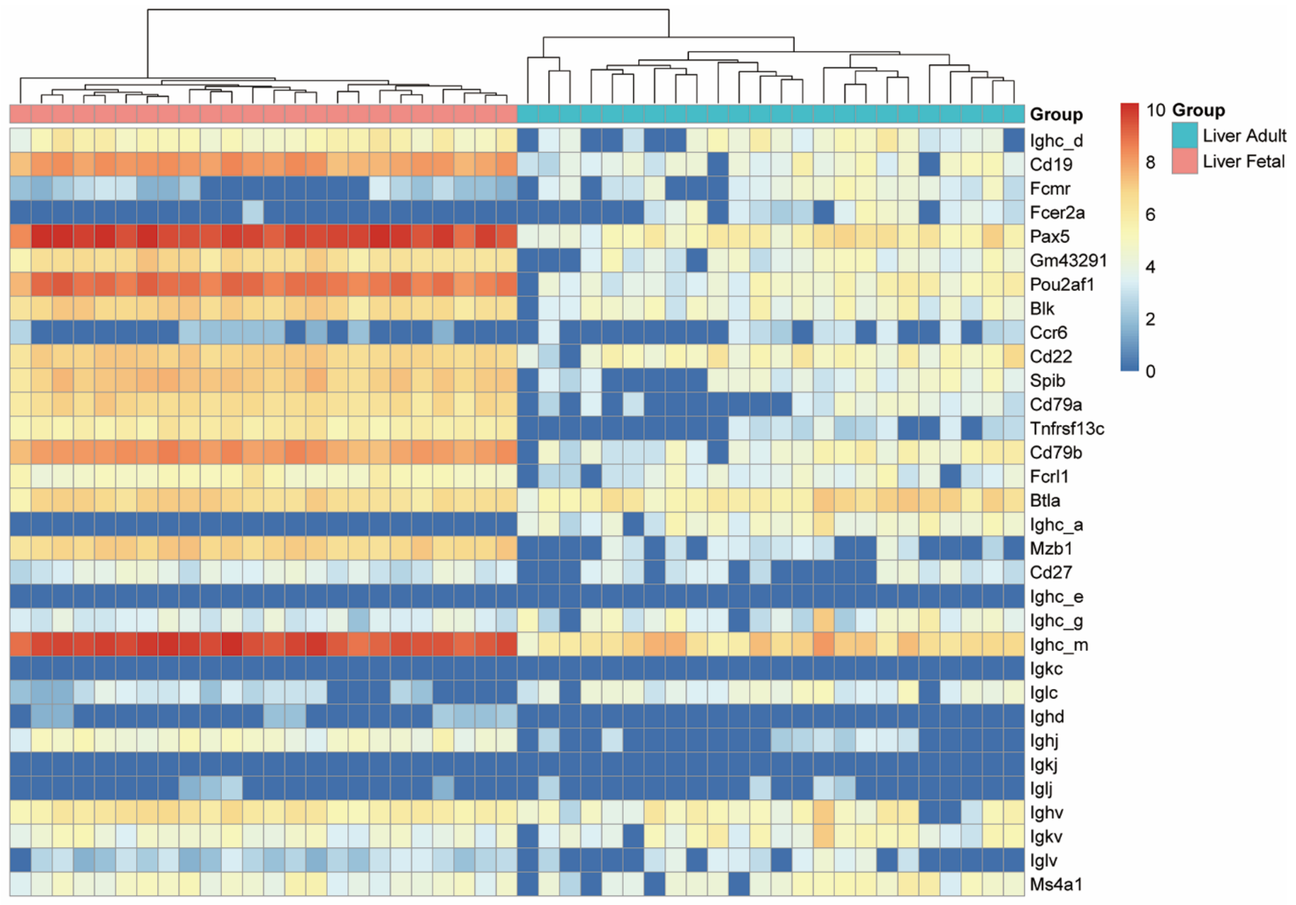

Supplementary Figure 6

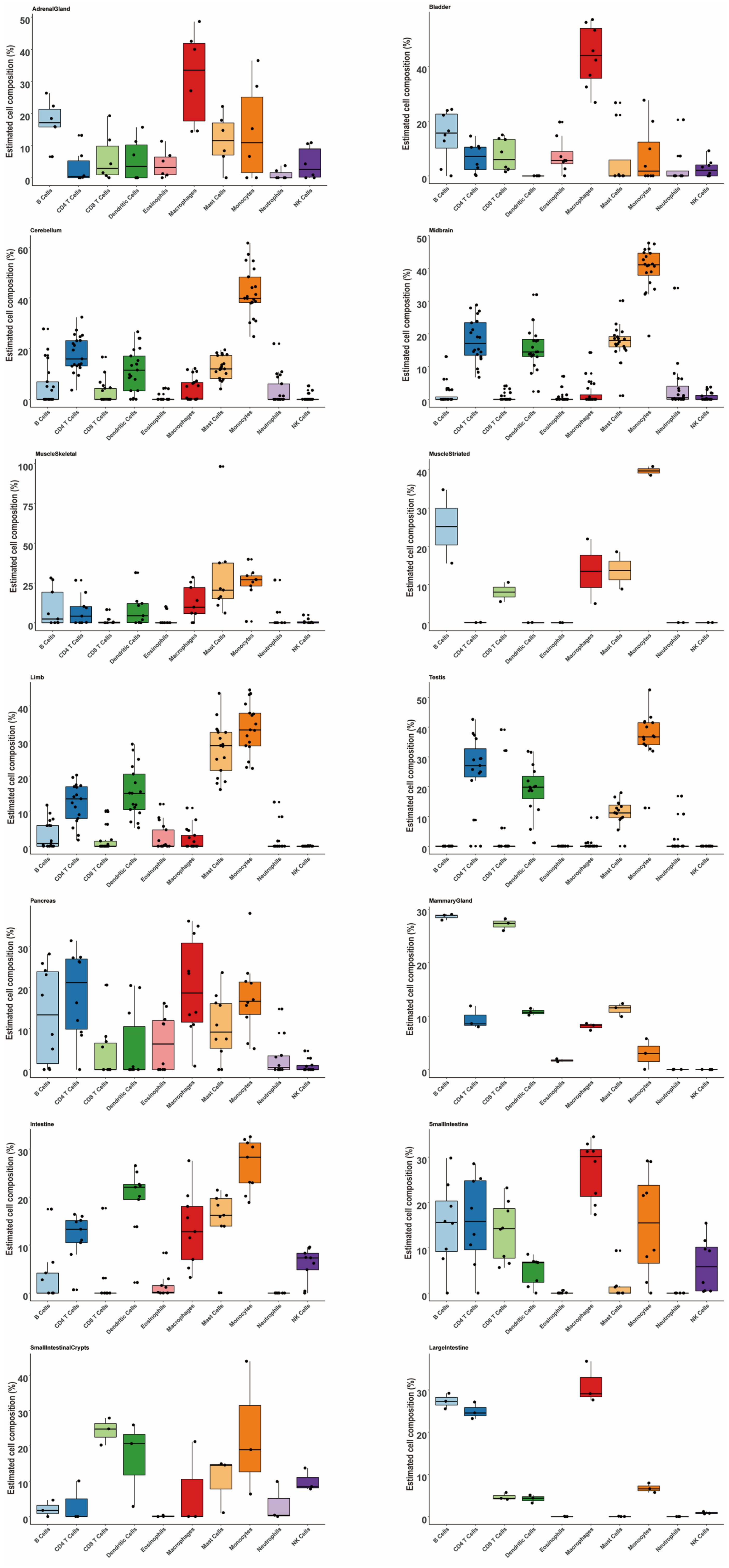

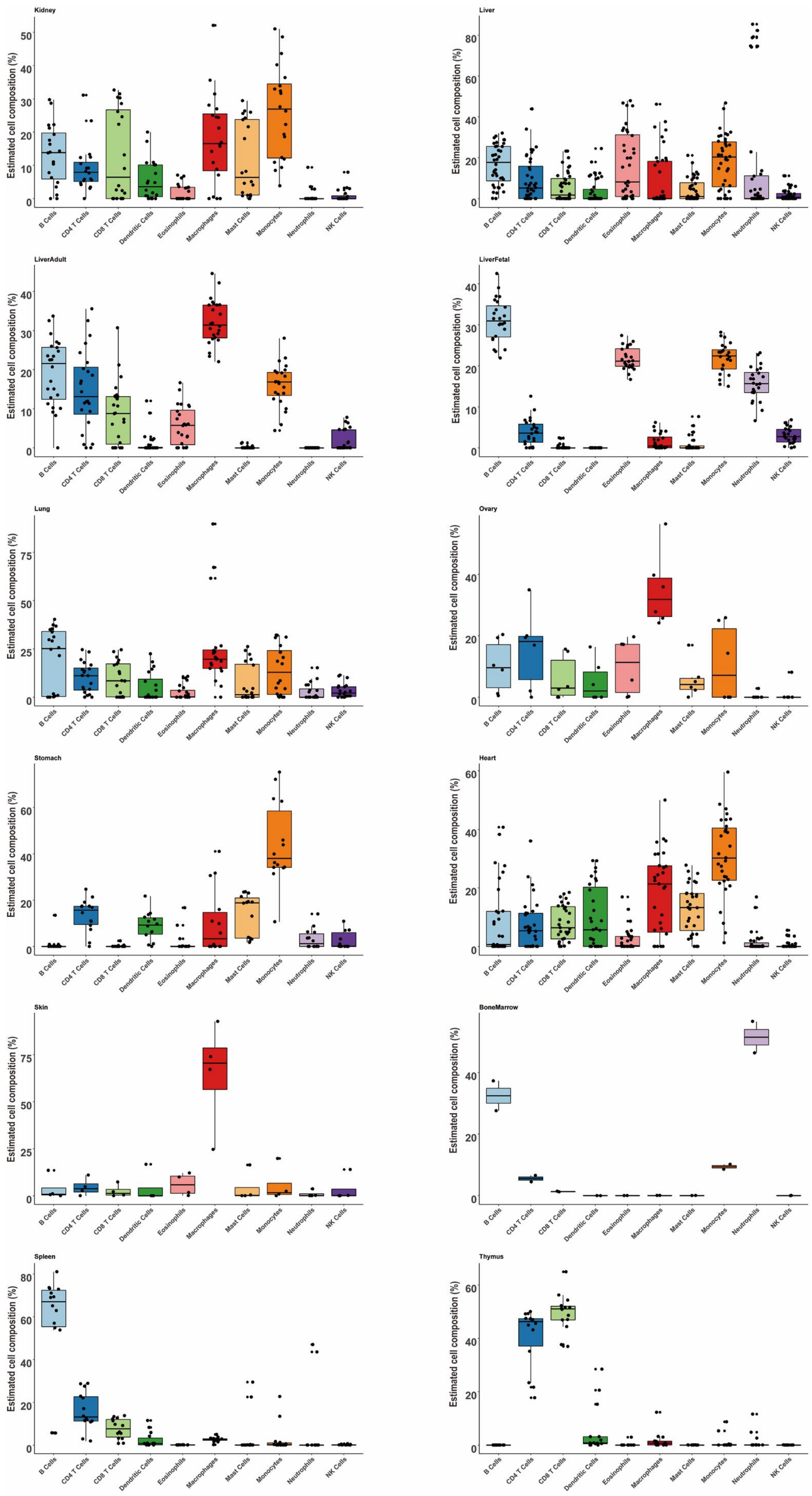
Kidney
Liver
LiverAdult
LiverFetal
Lung
Ovary
Stomach
Heart
Skin
BoneMarrow
Spleen
Thymus
Estimated cell composition (%)
B Cells
CD4 T Cells
CD8 T Cells
Dendritic Cells
Eosinophils
Macrophages
Mast Cells
Monocytes
Neutrophils
NK Cells

Supplementary Figure 7

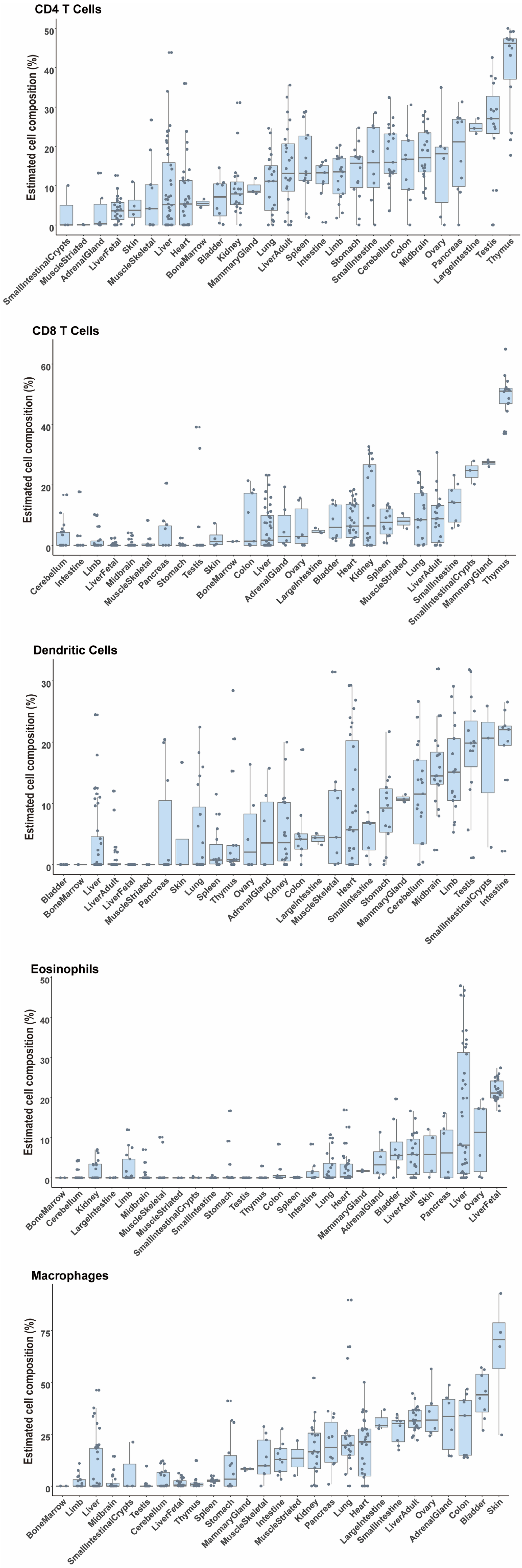

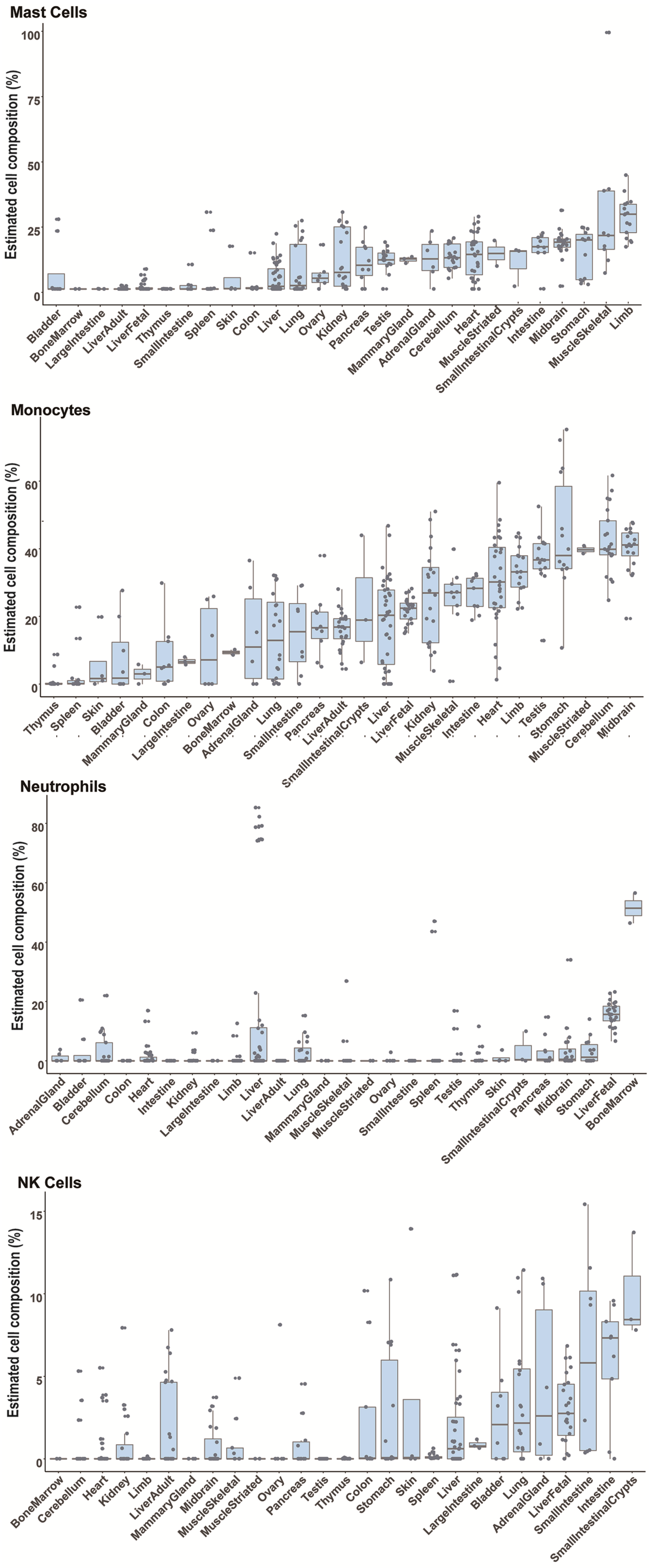
Mast Cells
Estimated cell composition (%)
100
75
50
25
0
Bladder
BoneMarrow
LargeIntestine
LiverAdult
LiverFetal
Thymus
SmallIntestine
Spleen
Skin
Colon
Liver
Lung
Ovary
Kidney
Pancreas
Testis
MammaryGland
AdrenalGland
Cerebellum
Heart
MuscleStriated
SmallIntestinalCrypts
Intestine
Midbrain
Stomach
MuscleSkeletal
Limb
Monocytes
Estimated cell composition (%)
60
40
20
0
Thymus
Spleen
Skin
Bladder
MammaryGland
Colon
LargeIntestine
Ovary
BoneMarrow
AdrenalGland
Lung
SmallIntestine
Pancreas
LiverAdult
SmallIntestinalCrypts
Liver
LiverFetal
Kidney
MuscleSkeletal
Intestine
Heart
Limb
Testis
Stomach
MuscleStriated
Cerebellum
Midbrain
Neutrophils
Estimated cell composition (%)
80
60
40
20
0
AdrenalGland
Bladder
Cerebellum
Colon
Heart
Intestine
Kidney
LargeIntestine
Limb
Liver
LiverAdult
Lung
MammaryGland
MuscleSkeletal
MuscleStriated
Ovary
SmallIntestine
Spleen
Testis
Thymus
Skin
SmallIntestinalCrypts
Pancreas
Midbrain
Stomach
LiverFetal
BoneMarrow
NK Cells
Estimated cell composition (%)
15
10
5
0
BoneMarrow
Cerebellum
Heart
Kidney
Limb
LiverAdult
MammaryGland
Midbrain
MuscleSkeletal
MuscleStriated
Ovary
Pancreas
Testis
Thymus
Colon
Stomach
Skin
Spleen
Liver
LargeIntestine
Bladder
Lung
AdrenalGland
LiverFetal
SmallIntestine
Intestine
SmallIntestinalCrypts

Supplementary Figure 8

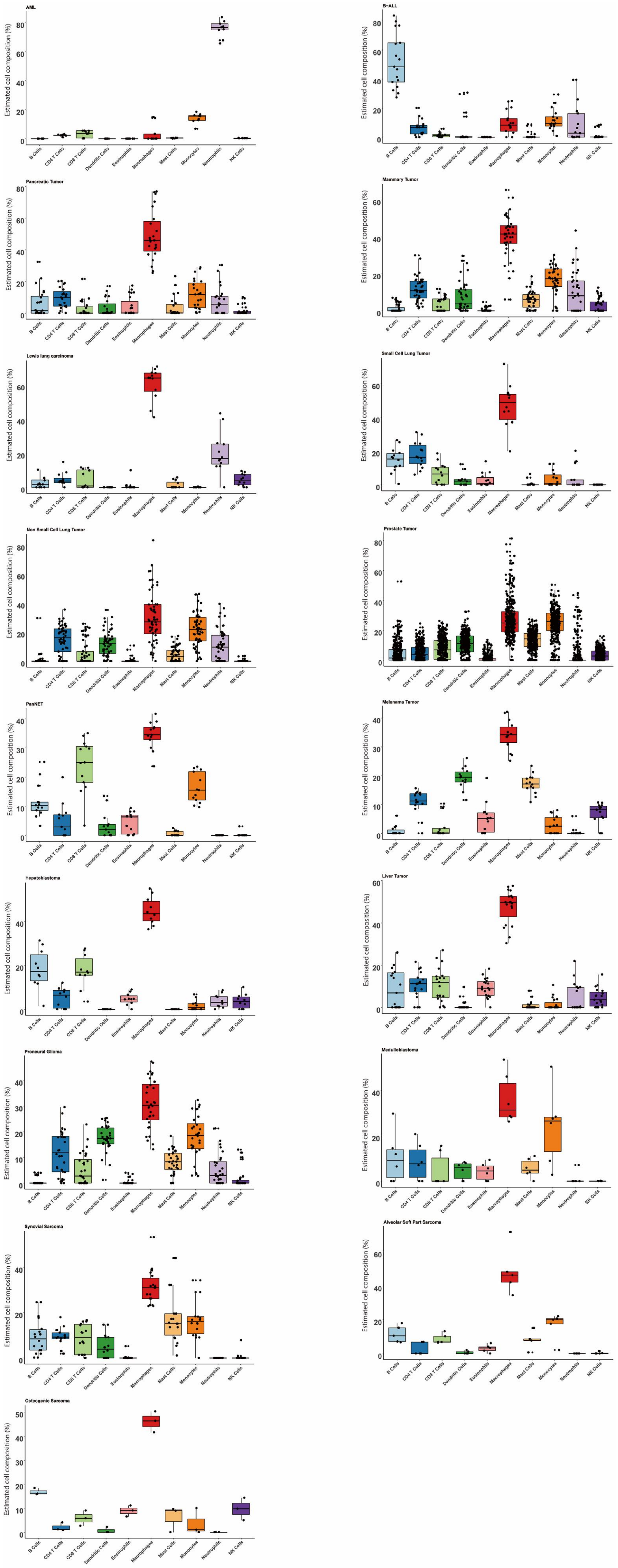

Supplementary Figure 9

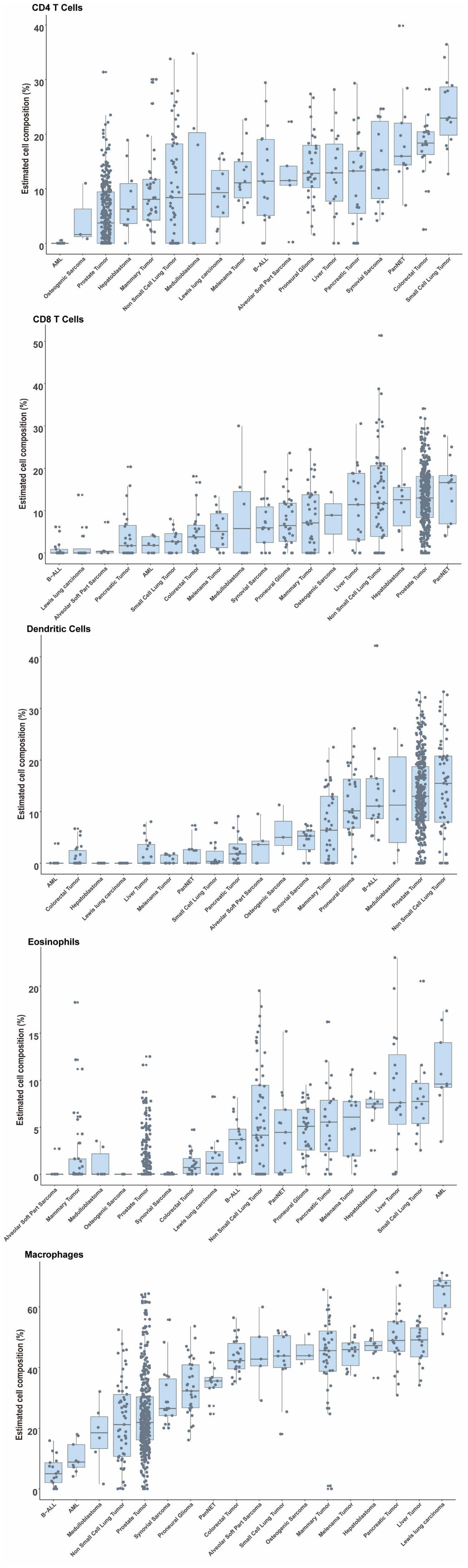

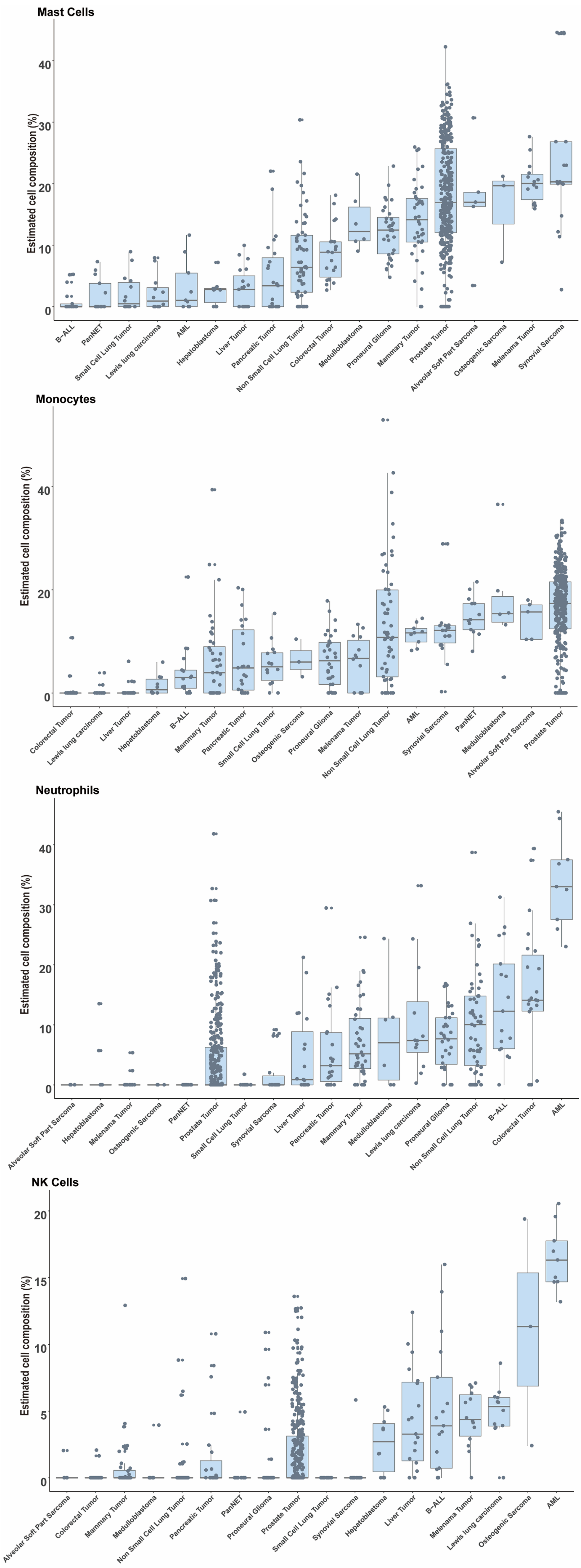

Mast Cells
Estimated cell composition (%)
0
10
20
30
40
B-ALL
PanNET
Small Cell Lung Tumor
Lewis lung carcinoma
AML
Hepatoblastoma
Liver Tumor
Pancreatic Tumor
Non Small Cell Lung Tumor
Colorectal Tumor
Medulloblastoma
Proneural Glioma
Mammary Tumor
Prostate Tumor
Alveolar Soft Part Sarcoma
Osteogenic Sarcoma
Melenama Tumor
Synovial Sarcoma
Monocytes
Estimated cell composition (%)
0
20
40
Colorectal Tumor
Lewis lung carcinoma
Liver Tumor
Hepatoblastoma
B-ALL
Mammary Tumor
Pancreatic Tumor
Small Cell Lung Tumor
Osteogenic Sarcoma
Proneural Glioma
Melenama Tumor
Non Small Cell Lung Tumor
AML
Synovial Sarcoma
PanNET
Medulloblastoma
Alveolar Soft Part Sarcoma
Prostate Tumor
Neutrophils
Estimated cell composition (%)
0
10
20
30
40
Alveolar Soft Part Sarcoma
Hepatoblastoma
Melenama Tumor
Osteogenic Sarcoma
PanNET
Prostate Tumor
Small Cell Lung Tumor
Synovial Sarcoma
Liver Tumor
Pancreatic Tumor
Mammary Tumor
Medulloblastoma
Lewis lung carcinoma
Proneural Glioma
Non Small Cell Lung Tumor
B-ALL
Colorectal Tumor
AML
NK Cells
Estimated cell composition (%)
0
5
10
15
20
Alveolar Soft Part Sarcoma
Colorectal Tumor
Mammary Tumor
Medulloblastoma
Non Small Cell Lung Tumor
Pancreatic Tumor
PanNET
Proneural Glioma
Prostate Tumor
Small Cell Lung Tumor
Synovial Sarcoma
Hepatoblastoma
Liver Tumor
B-ALL
Melenama Tumor
Lewis lung carcinoma
Osteogenic Sarcoma
AML
