# Supplementary material for: seq-ImmuCC: Cell-Centric View of Tissue Transcriptome Measuring Cellular Compositions of Immune Microenvironment From Mouse RNA-Seq Data
Source: Front Immunol. 2018 Jun 5;9:1286. doi: 10.3389/fimmu.2018.01286 (PMC5996037; doi:10.3389/fimmu.2018.01286)
Supplement: Supplementary file 3 [file data_sheet_1.docx]

**Supplementary Figure Legends**

**Supplementary Figure 1.** Schematic of the ImmuCC model construction.

**Supplementary Figure 2.** PCA of 162 selected genes in 286 enriched immune cell data.

**Supplementary Figure 3.** Comparison of the RNA-Seq training and testing models with the flow

cytometry for Granulo-monocytic cells, CD4 T cells, CD8 T cells, and B cells in the bone marrow, lymph nodes, and spleen.

**Supplementary Figure 4.** Boxplot of IgA expression in 27 mouse tissues.

**Supplementary Figure 5.** Heatmap for the expression profile of B cell-specific genes in the fetal liver and the adult liver.

**Supplementary Figure 6.** Inferred proportions of 10 immune cells in 26 mouse tissues.

**Supplementary Figure 7.** Distribution of CD4 T cells, CD8 T cells, macrophages, monocytes, neutrophils, mast cells, eosinophils, dendritic cells and natural killer cells proportion across 27 mouse tissues.

**Supplementary Figure 8.** Inferred proportions of 10 immune cells in 17 mouse tumor tissues.

**Supplementary Figure 9.** Distribution of CD4 T cells, CD8 T cells, macrophages, monocytes, neutrophils, mast cells, eosinophils, dendritic cells and natural killer cells proportion across 18 mouse tumor tissues.

**Supplementary Table Legends**

**Supplementary Table 1.** Immune cell data sets collected from the public database and the inferred immune proportion in both the normal tissue and the tumor tissues
